# Syncytiotrophoblast differentiation defects drive ER stress and reprogram placental PSG1 glycosylation in preeclampsia

**DOI:** 10.1186/s43556-026-00634-x

**Published:** 2026-10-30

**Authors:** Charlotte Harms, Yiru Wang, James Warren, Julia Baumeister, Fabian Higel, Cristina Antich, Marc Ferrer, Jennifer J. Hill, Mariana G. Garcia, Alexandra Gellhaus, Nándor Gábor Than, Florian Herse, Stefan Verlohren, Gabriela S. Dveksler, Sandra M. Blois

**Affiliations:** 1 Department of Obstetrics and Fetal Medicine, University Medical Center Hamburg-Eppendorf, Hamburg, Germany; 2 Glyco-HAM, a Cooperation of Universität Hamburg, Technology Platform Mass Spectrometry and University Medical Center Hamburg-Eppendorf, Hamburg, Germany; 3 Department of Pathology, Uniformed Services University of the Health Sciences, Bethesda, MD 20817 USA; 4 Global Development CMC Biologicals, Boehringer Ingelheim Pharma GmbH & Co. KG, Biberach an Der Riss, Germany; 5 3D Tissue Bioprinting Laboratory, National Center for Advancing Translational Sciences, National Institute of Sciences, Bethesda, MD USA; 6 Human Health Therapeutics, National Research Council Canada, Ottawa, ON Canada; 7 Ottawa Institute of Systems Biology, Ottawa, ON Canada; 8 Department of Gynecology and Obstetrics, University of Duisburg-Essen, Essen, Germany; 9 Systems Biology of Reproduction Research Group, Institute of Molecular Life Sciences, HUN-REN Research Centre for Natural Sciences, Budapest, Hungary; 10 Department of Obstetrics and Gynecology, School of Medicine, Semmelweis University, Budapest, Hungary; 11 Maternity Private Clinic of Obstetrics and Gynecology, Budapest, Hungary; 12 Genesis Theranostix Group, Budapest, Hungary; 13 Experimental and Clinical Research Center, Max‐Delbrück‐Center for Molecular Medicine in the Helmholtz Association and the Charité ‐ Universitätsmedizin Berlin, Berlin, Germany; 14 Max-Delbrück-Center for Molecular Medicine in the Helmholtz Association, Berlin, Germany

**Keywords:** PSG1, Preeclampsia, STB differentiation, *N*-glycosylation, ER stress

## Abstract

During pregnancy, placenta-derived proteins such as pregnancy-specific glycoprotein 1 (PSG1) enter the maternal circulation, providing a window into placental health and enabling liquid biopsy strategies for monitoring pregnancy disorders. PSG1, a highly glycosylated protein secreted by syncytiotrophoblast (STB) cells, reflects trophoblast differentiation status. Circulating PSG1 levels are reduced in early-onset preeclampsia (PE), but how PSG1 glycosylation is altered in this condition remains unclear. Here, we show by liquid chromatography-tandem mass spectrometry that native PSG1 purified from healthy and early-onset PE pregnancies carries an *O*-linked glycan in its N domain in addition to diverse *N*-linked glycans. In PE, PSG1 *N*-glycans displayed a shift toward increased oligomannose structures and reduced sialylation. Induction of endoplasmic reticulum (ER) stress in primary STBs altered the glycosylation enzyme machinery, and single-nucleus transcriptomic data indicated a reduced contribution of mature STB populations in PE, consistent with an immature STB-like glycoprotein signature. Altered *N*-glycosylation increased binding to mannose and asialoglycoprotein receptors and cellular uptake, suggesting enhanced hepatic clearance as a possible explanation for reduced circulating PSG1 levels in PE. Our data link ER stress during STB differentiation to altered glycoprotein maturation and identify PSG1 glycosylation as a readout of a stress-impaired STB in PE. Glycan features of circulating placental proteins could therefore provide information beyond protein levels alone and complement liquid biopsy approaches.

**Supplementary Information:**

The online version contains supplementary material available at https://doi.org/10.1186/s43556-026-00634-x.

## Introduction

The developing placenta secretes proteins into maternal circulation as early as the first trimester, which regulate several biological processes required for a healthy pregnancy [1]. Among these are the members of the pregnancy-specific glycoprotein (PSG) family, originally designated Schwangerschafts Protein 1 (SP1), that are mainly expressed by syncytiotrophoblast (STB) cells [2–6]. PSG1 is the best studied of the 10 PSG family members and exhibits anti-inflammatory [7] and pro-angiogenic functions [8], and interacts with galectins-1 (gal-1) and -9, immunoregulatory glycan-binding proteins that participate in placental development [9, 10]. Notably, altered levels of PSG1 have been associated with pregnancy complications, including reduced levels during preeclampsia (PE) [6, 11–14].

PE is a multisystemic obstetrical syndrome that affects 2%–5% of all pregnancies, leading to the death of 76 000 women and 500 000 babies every year [15]. PE can be classified into early-onset, which is defined by a delivery before 34 weeks, and typically associated with more severe outcomes, and late-onset, with a delivery after 34 weeks of gestation [15]. It has been demonstrated that mainly early-onset PE is associated with increased placental oxidative stress, elevated levels of reactive oxygen species, and endoplasmic reticulum (ER) stress, which contribute to the pathogenesis of the disease [16–20]. These stressors give rise to an anti-angiogenic and inflammatory milieu and are accompanied by an altered glycosylation pattern in the STB [21, 22].

Protein post-translational modifications (PTMs) increase the functional diversity of the proteome. Glycosylation is one of the most abundant PTMs and is known to impact cell–cell interactions, protein conformation, protein half-life in circulation, and immunomodulatory activity [22–26]. Previous reports indicated that glycosylation is altered in early-onset PE, potentially affecting regulatory functions that influence healthy pregnancy dynamics [27]. PSG1 is composed of four domains (designated as N, A1, A2, and B2) with a total of seven *N-*glycosylation sites located in the N, A1, and A2 domains [9]. Previous reports indicated that the carbohydrate content of PSG1 accounts for approximately 30% of its molecular weight [28, 29] and we recently analysed its global *N-*glycosylation by a mass spectrometric glycomic and glycoproteomic approach in PSG1 obtained from pooled serum of healthy pregnant women. In these studies, we identified the presence of complex *N-*glycans containing poly LacNac epitopes with α2,3-sialyation at 4 sites [9].

To date, there is no information regarding (1) PSG1 *O-*glycosylation and (2) changes in PSG1 glycosylation in PE. The present study used nano-liquid chromatography-tandem mass spectrometry (nanoLC–MS/MS) to analyse site-specific glycosylation of PSG1 in normal pregnancy and early-onset PE. Notably, we identified an *O-*linked glycan in the N domain of PSG1. Our findings further revealed alterations in the *N-*glycosylation of PSG1 from early-onset PE pregnancies, characterized by elevated oligomannose and reduced sialic acid abundance, compared to healthy pregnancies. Induction of ER stress in primary STBs, as observed in PE, altered the enzymatic machinery involved in this PTM. In line with these findings, analysis of single-nucleus RNA (snRNA) sequencing data [30] indicated a shift in PSG1 expression towards immature STBs in PE, accompanied by higher ER stress levels and altered glycosyltransferase expression. The observed changes in PSG1 glycosylation increased its interaction with hepatic clearance receptors and enhanced its cellular uptake by glycoprotein receptor-expressing cells. We therefore propose that changes in PSG1 *N-*glycosylation may contribute to reduced PSG1 serum levels in early-onset PE, whereas the role of its *O-*linked glycan requires further investigation.

## Results

### Native PSG1 carries *O-*linked glycans

**Fig. 1:**
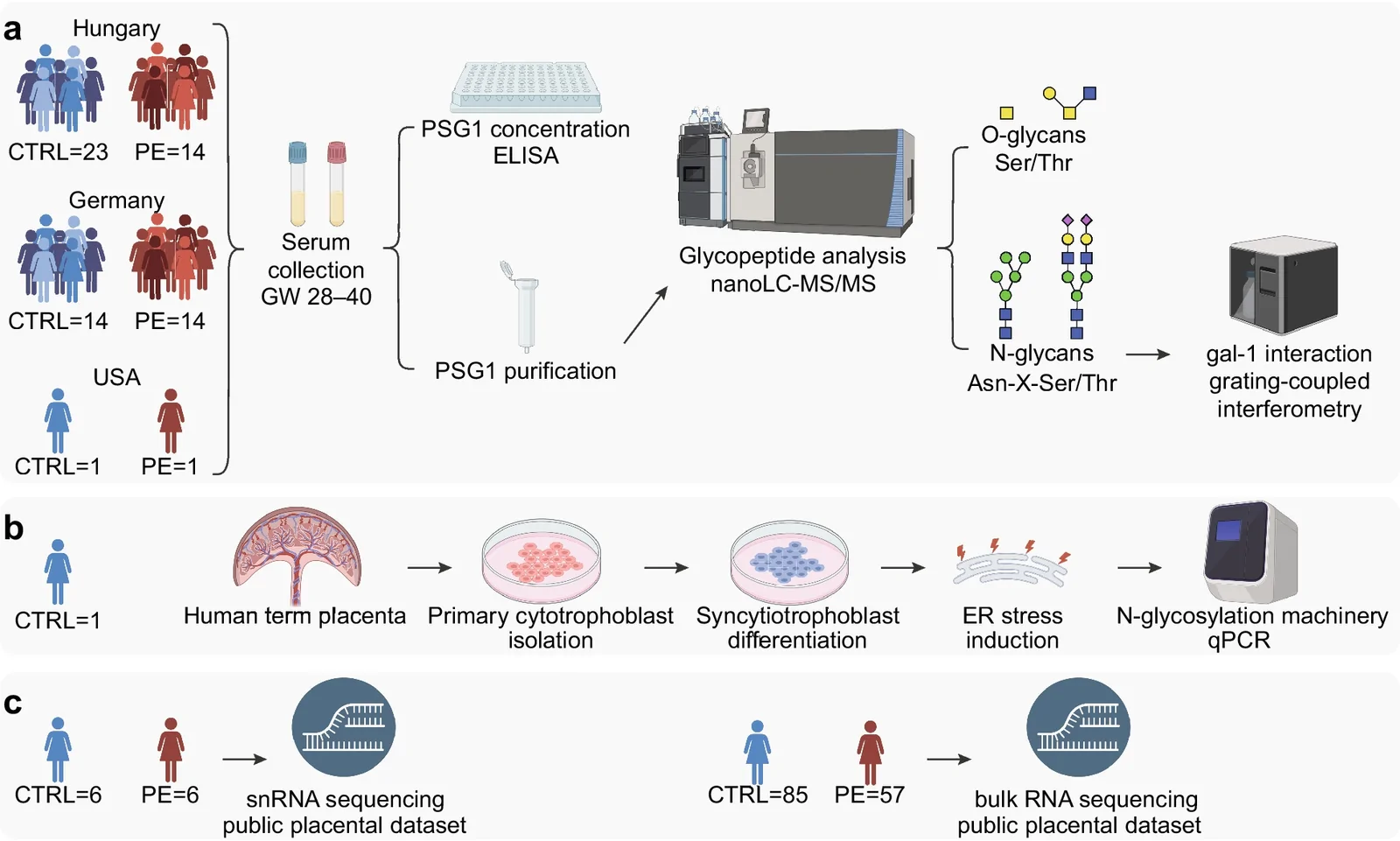
Flowchart representing the study design.** a** Experimental outline and studies performed with the indicated human cohorts. **b** Workflow of the experiment performed in vitro using term placenta cytotrophoblasts. **c** Analysis of gene expression in two previously published RNA sequencing datasets. Abbreviations: CTRL: native PSG1 from healthy pregnant women; PE: preeclamptic pregnancy; GW: gestational week; gal-1: galectin-1. Partially created in BioRender. Harms, C. (2026) https://BioRender.com/v4ygiwx

An overview of the study design is presented in Fig. 1. We performed peptide-specific *O-*glycan analysis of native PSG1 from three cohorts (Table 1), using nanoLC–MS/MS to determine whether native PSG1 is decorated with *O-*linked glycans. In PSG1 from healthy pregnancies (PSG1^CTRL^) and those complicated with early-onset PE (PSG1^PE^), we identified one *O-*glycosylated peptide within the N domain (Table 2). The corresponding MS/MS spectra are shown in Fig. S1a and S1b. This tryptic peptide is *O-*glycosylated with a HexNAc(1)Hex(1) (core 1/T-antigen) and located following PSG1’s 34-amino-acid signal sequence. The binding of PNA, a lectin that specifically binds to core 1 and core 2 *O-*glycans [31], to native PSG1 from control and PE pregnancies confirmed the presence of *O-*glycosylation in this protein, as shown in Fig. S2a and S2b.

**Table 1:** Clinical characteristics of the patients. Summary of clinical parameters including age, body mass index (BMI), systolic and diastolic blood pressure, presence of proteinuria, newborn birth weight, placental weight, incidence of intrauterine growth restriction (IUGR), and neonatal sex. Statistical comparisons were conducted between control and early-onset preeclampsia (PE) groups within each cohort: Hungary, Germany, and the USA

Clinical Parameter	Hungary			Germany			USA	
	**Term control (n = 23)**	**Early-onset PE (n = 14)**	**P value**	**Term control (n = 14)**	**Early-onset PE (n = 14)**	**P value**	**Term control (n = 1)**	**Early-onset PE (n = 1)**
Age [year]^1^	32 (21, 38)	30 (23, 38)	0.58	34 (26, 46)	32.5 (23, 40)	0.31	-	31
Body mass index [kg/m^2^]^1^	26.08 (20.20, 34.16)	24.34 (19.94, 34.25)	0.25	34 (25, 39)	30 (24, 37)	0.05	-	-
Systolic blood pressure [Hgmm]^1^	120 (100, 144)	160 (136, 180)	< 0.001^***^	120 (85, 133)	147.5 (111, 162)	< 0.001^***^	-	severe
Diastolic blood pressure [Hgmm]^1^	78 (60, 96)	100 (88, 110)	< 0.001^***^	78 (50, 90)	87.5 (75, 105)	0.008^**^	-	severe
Proteinuria	No	Yes	-	Yes (3), No (5) 0 (0, 0.55)	Yes (12) 1.135 (0.22, 12.81)	- < 0.001^***^	No	Yes
Newborn weight [g]^1^	3440 (2860, 4810)	1010 (540, 1920)	< 0.001^***^	2845 (2220, 3860)	1070 (435, 2310)	< 0.001^***^	-	-
Placenta weight [g]^1^	545 (262, 750)	211 (116, 305)	< 0.001^***^	-	-	-	-	-
Intrauterine growth restriction	No	Yes (11), No (3)	-	No	Yes (12), No (2)	-	No	Yes
Sex male/female	10/13	7/7	-	5/7	6/8	-	1/0	1/0
Gestational age [weeks]^2^	38.86 ± 0.62	30.14 ± 4.29	< 0.001^***^	28.44 ± 6.30	28.63 ± 3.31	0.54	39.14	34.00
PSG1 level [µg/mL]^1^	17.35 (1.55, 90.76)	5.15 (0.82, 10.98)	< 0.001^***^	1.84 (0.42, 11.86)	2.41 (0.03, 24.78)	0.45	14.00	5.00

^1^Values are presented as median and range (minimum, maximum)

^2^Values are presented as mean ± SD

Significant differences are indicated by * *p* < 0.05, ** *p* < 0.01, *** *p* < 0.001 for each variable. Significant differences were tested by unpaired parametric Student’s t-test or Mann–Whitney test

**Table 2:** *O*-glycosylation of native PSG1. Overview of identified *O*-glycopeptides from tryptic digests of PSG1 analyzed by mass spectrometry. Glycopeptide sequences are shown using the one-letter amino acid code

PSG1	Observed m/z	Theoretical m/z	Delta mass [Da]	Delta mass [ppm]	Charge	Residue	Domain	Glycopeptide	Composition % mass
CTRL	982.486	982.483	0.0025	2.5446	2	35–49	N	QVTIEAEPTKVSEGK	HexNAc(1)Hex(1) % 365.1322
PE	982.483	982.483	0.0000	0.0000	2	35–49	N	QVTIEAEPTKVSEGK	HexNAc(1)Hex(1) % 365.1322

Abbreviations: CTRL: native PSG1 from healthy pregnant women; PE: native PSG1 from preeclamptic pregnant women; m/z: mass-to-charge ratio; HexNAc: N-acetylhexosamine; Hex: hexose

### PSG1 *N-*glycosylation differs between normal and early-onset PE pregnancies

The binding of PSG1 to gal-1 and -9 is influenced by its *N-*glycosylation [9, 10]. To determine whether *N-*glycosylation of PSG1 differs between healthy and PE pregnancies (Table 1), *N*-glycopeptides were analysed by a mass spectrometry-based approach. In native PSG1, *N-*glycosylation was found in six of the seven *N-*glycosylation sites. These sites are located in the N (N61, N104, and N111) and the A2 domains (N259 and N268, and N303). We did not detect the *N-*glycopeptide (N199) located in the A1 domain in any of the native PSG1 samples digested with trypsin or chymotrypsin. Full glycosylation site occupancy (100%) was observed at N61, N104, and N268 for both native PSG1 variants (PSG1^CTRL^ and PSG1^PE^). Partial occupancy was detected at other sites: N111 showed 87.3% in PSG1^CTRL^ and 76.2% in PSG1^PE^; N259 displayed 83.2% in PSG1^CTRL^ and 88.6% in PSG1^PE^; and N303 exhibited 99.4% in PSG1^CTRL^ and 99.5% in PSG1^PE^. It is important to note, however, that site-occupancy estimates may be biased due to differences in MS-based response factors between glycosylated and non-glycosylated peptides.

**Fig. 2:**
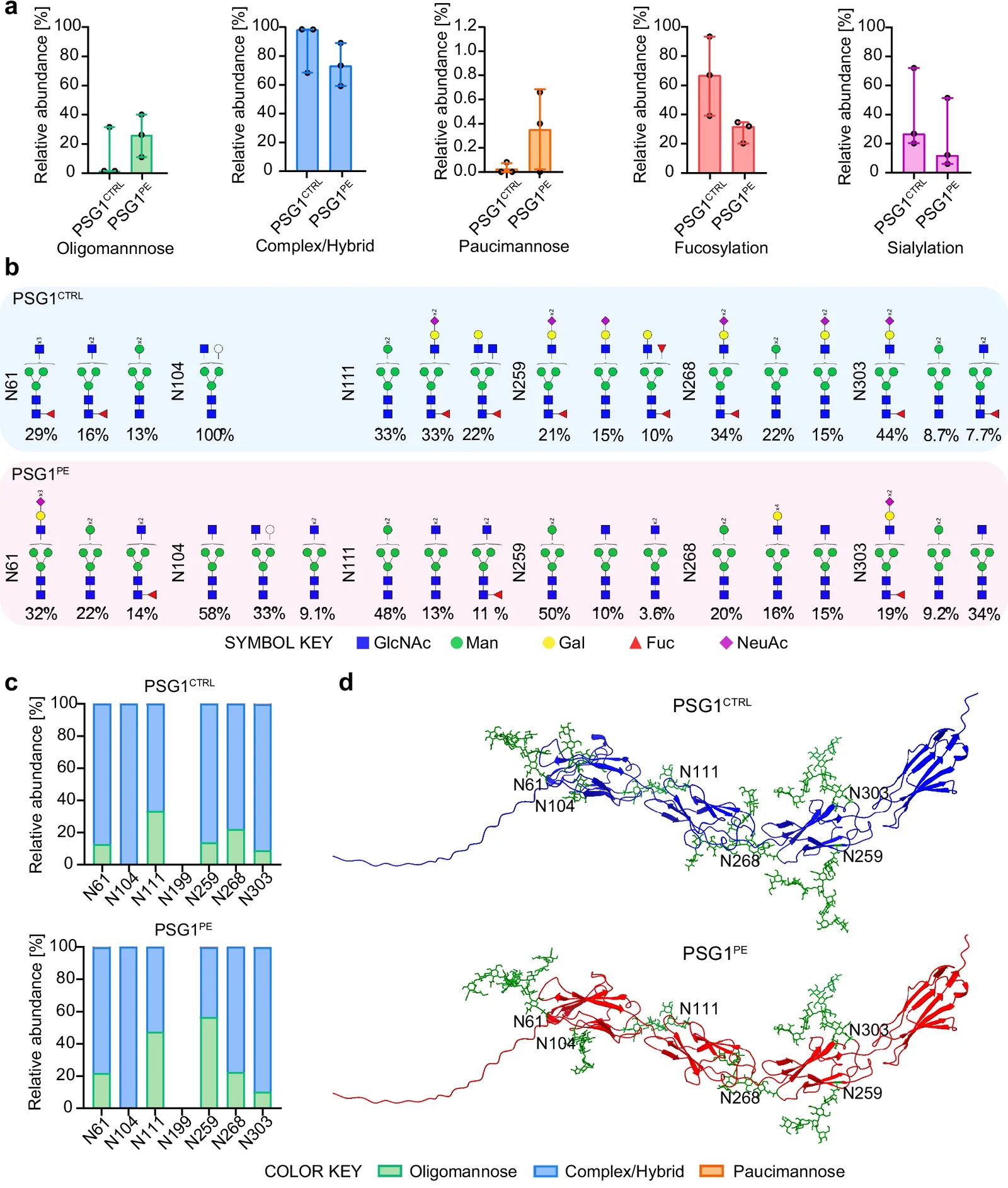
*N*-glycosylation pattern of native PSG1 as determined by nanoLC–MS/MS. **a** Relative abundance of glycosylation traits on *N*-glycopeptides represented as percentage of the total abundance of glycopeptides. Data of native PSG1’s *N*-glycosylation from healthy and preeclamptic pregnant women from three cohorts. Bar graphs represent the median ± range, and differences between conditions were assessed using the Wilcoxon signed-rank test.** b** Visualization of the most abundant *N-*glycan structures with their relative abundance at each *N*-glycosylation site of native PSG1 isolated from healthy pregnant women (upper) and from pregnancies complicated with preeclampsia (lower). **c** Site-specific relative abundance of *N-*glycan classes in native PSG1 from healthy (upper) and preeclamptic pregnant women (lower). Bar graphs indicate the mean. Glycan classes are indicated by colours as represented in the color key. **d** Computational modelling of PSG1 protein structures with the most abundant glycan structure at each *N-*glycosylation site for native PSG1^CTRL^ (upper) and PSG1^PE^ (lower) as analysed by LC–MS/MS (Generated using AlphaFold and GLYCAM 3D [32, 33])

Interestingly, we identified an increase in the relative abundance of oligomannose *N-*glycans in PSG1^PE^ (26.2% [11.0% – 40.1%]; median [range]) compared to PSG1^CTRL^ (1.54% [1.49% – 31.5%]) and a decrease in complex-type *N-*glycans in PSG1^PE^ (73.4% [59.2% – 89.0%]) compared to PSG1^CTRL^ (98.5% [68.4% – 98.5%]). In addition, paucimannose glycans were increased in PSG1^PE^ (0.40% [0.00% – 0.66%]) compared to PSG1^CTRL^ (0.00% [0.00% – 0.80%]). Furthermore, 26.9% [20.3% – 72.1%] of PSG1^CTRL^ and only 12.2% [6.12% – 51.5%] of PSG1^PE^ *N-*glycans were sialylated. The abundance of fucosylated *N-*glycans was lower in PSG1^PE^, with only 32.0% [20.3% – 34.7%] compared to 66.9% [39.1% – 93.4%] in PSG1^CTRL^ (Fig. 2a). While the difference in glycosylation did not reach statistical significance across the three cohorts analysed (Wilcoxon signed-rank test), this likely reflects the high heterogeneity among groups and the limited sample size. In PSG1^CTRL^, biantennary *N-*glycans fully capped by NeuAc counted as the most abundant structures in four out of the six detected sites, which agrees with our previously published observations [9] showing that the most abundant glycans were fully capped biantennary glycans carrying terminal NeuAc with and without fucose (Fig. 2b). In PSG1^PE^, however, the most abundant structure in three of the six sites was an oligomannose glycan (Man5GlcNAc2). In native PSG1 from both healthy controls and early-onset PE patients, the relative abundance of oligomannose glycans was increased at the sites N111, 259, and 268 compared to the terminal sites at N61 and 303 (Fig. 2c). No oligomannose structures were identified at N104. The computational molecular structure for PSG1^CTRL^ and PSG1^PE^, generated using the AlphaFold model P11464 and the GLYCAM 3D glycoprotein structure prediction tool [32, 33], visualizes the most abundant glycan at each site to illustrate the identified glycosylation changes within their structural context (Fig. 2d). Furthermore, we performed a sialic acid linkage analysis, as this minor change in glycan structure can influence ligand interaction, including gal-1 binding [34]. After cleavage of α2,3-linked sialic acid, only one *N-*glycopeptide containing a sialic acid, a biantennary mono-sialylated and fucosylated glycan at N111, was identified in PSG1^PE^ (Table S1).

### Recombinant PSG1 *N-*glycosylation mirrors native PSG1 *N-*glycosylation patterns

We generated recombinant PSG1 in CHO and 293 cells as described in the materials and methods section. In both recombinants, *N-*glycosylated peptides were found for all seven sites in the N, A1, and A2 domains. A comparison of the *N-*glycans in PSG1^CHO^ and PSG1^293^ revealed that PSG1^293^ had lower levels of complex *N-*glycans (PSG1^CHO^: 94.24% vs. PSG1^293^: 77.75%) while it was enriched in oligomannose *N-*glycans (PSG1^CHO^: 4.56% vs. PSG1^293^: 21.92%). Paucimannose structures made up 1.2% of PSG1^CHO^ and 0.33% of PSG1^293^ *N-*glycans. Fucosylation of both recombinants was similar, with 38.31% of PSG1^CHO^ and 38.02% of PSG1^293^ glycans being fucosylated. The relative abundance of terminal sialic acid was lower in recombinant than in native PSG1 and also differed between recombinants, with 10.2% for PSG1^CHO^ and only 5.89% for PSG1^293^ (Fig. S3a). All sialic acids were identified as α2,3-linked, as no sialylated glycans were detected after Sialidase S treatment. Interestingly, the differences in the *N-*glycosylation of oligomannose, complex, and sialylated glycans showed the same trends when comparing PSG1^CTRL^ to PSG1^PE^ and PSG1^CHO^ to PSG1^293^. The relative abundance of each glycosylation trait across the whole protein is visualized in Fig. S3a and the three most abundant *N-*glycan structures with their mean relative abundance are shown in Fig. S3b.

### ER stress impairs *N-*glycan maturation

**Fig. 3:**
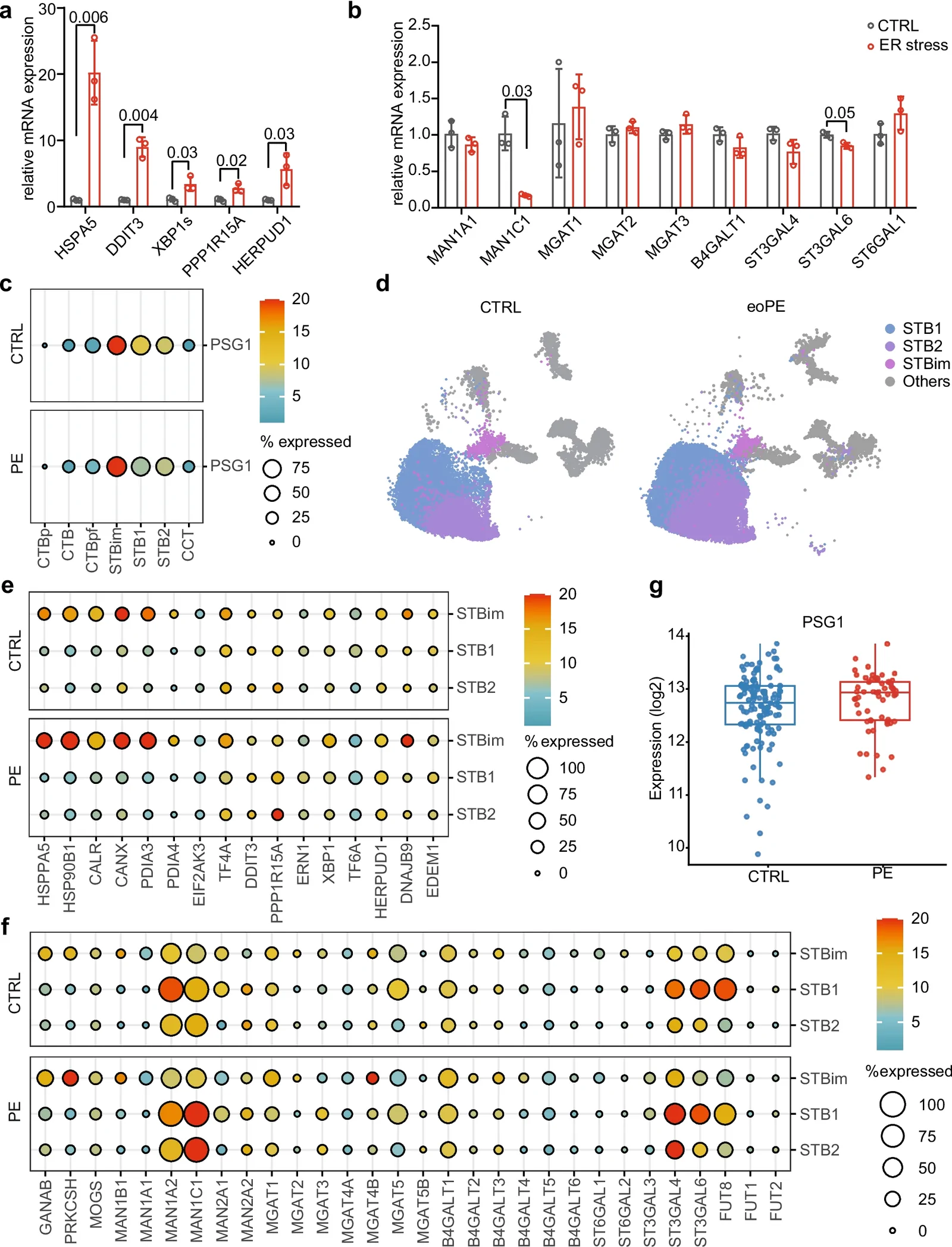
ER stress disrupts *N*-glycan machinery expression in primary syncytiotrophoblasts and is linked to syncytiotrophoblast immaturity in preeclampsia. **a** ER stress markers in primary STBs analysed by qPCR (mean ± SD; multiple t-tests with Benjamini–Hochberg correction). **b** Enzyme machinery responsible for *N-*glycan synthesis in primary STBs analysed by qPCR (mean ± SD; multiple t-tests with Benjamini–Hochberg correction). **c** PSG1 expression in different types and stages of trophoblast cells in control and early-onset preeclampsia placentas identified in a previously published snRNA sequencing dataset (EGAS00001005681).** d** Cell type annotation and distribution in control and early-onset preeclampsia placentas identified in the previously published snRNA sequencing dataset.** e** ER stress marker expression in different stages of STB cells in control and early-onset preeclampsia placentas identified in the previously published snRNA sequencing dataset.** f** ER stress marker expression in different stages of STB cells in control and early-onset preeclampsia placentas identified in the previously published snRNA sequencing dataset.** g** Bulk placental RNA expression of PSG1 in healthy and early-onset preeclamptic pregnancies (GSE75010 subset, mean ± SD). Statistical analysis was performed by linear regression adjusted for dataset of origin and gestational age. Abbreviations: ER: Endoplasmic Reticulum; STB: syncytiotrophoblast; im: immature; CCT: cell column trophoblast; CTB: cytotrophoblast; p: proliferating; pf: pre-fusion

PSG1 is predominantly expressed by STBs [5, 6]. To investigate how ER stress affects *N-*glycan biosynthesis, we analysed the expression of key *N-*glycosylation enzymes in primary STBs following thapsigargin treatment. ER stress induction was confirmed by significant upregulation of ER stress markers after 24 h of treatment (Fig. 3a). Among nine key placental glycosyltransferases analysed by RT-qPCR, we observed a significant downregulation of MAN1C1 and ST3GAL6, while there were non-significant differences in the expression of the other enzymes analysed (Fig. 3b).

### Maturation of PSG1-expressing trophoblasts influences glycosylation patterns

To analyse the expression patterns of PSG1 in human STBs from control and early-onset PE placentas, we consulted publicly available snRNA sequencing data from Nonn et al. (2025), accessible at the European Genome-Phenome Archive under the accession number EGAS00001005681 (Fig. 3c) [30]. The previously processed data revealed three different maturation stages of STBs (STBim < STB1 < STB2; Fig. 3d). Figure 3c shows the PSG1 expression patterns across all trophoblast cells, with STBim having the highest expression, followed by STB1. In STB1, PSG1 expression was significantly reduced in early-onset PE placentas compared to controls (log2FC = −0.51, adjusted p = 3.06 × 10^–58^, Bonferroni-corrected LR test) [30]. In both groups, STBim expressed higher levels of ER stress markers (Fig. 3e) and lower levels of most glycosyltransferases (Fig. 3f). Specifically, the expression of α2,3-sialyltransferases and the expression of the major α-mannosidases were reduced in the STBim compared to the more mature STBs. Additionally, analysis of a previously published combined microarray bulk placental RNA-sequencing dataset even revealed a significant increase in PSG1 mRNA expression in placentas from early-onset PE compared to controls after adjusting for gestational age and dataset (β = 0.29, p = 0.016; Fig. 3g).

### Observed changes in *N-*glycosylation do not affect the PSG1–galectin-1 interaction

To further evaluate the relevance of the identified changes in PSG1 glycosylation, we analysed the binding affinity of native and recombinant PSG1 preparations to gal-1 by grating-coupled interferometry. Across the different PSG1s, we observed comparable interaction kinetics with gal-1, as our results only showed negligible variance and a similar dissociation constant between 1.6 to 1.8 μM (KD) (Fig. S4a–d). However, immobilized PSG1^PE^ showed a slight trend towards an increased KD compared to PSG1^CTRL^. As described before [9], both interactions could be inhibited by adding lactose but not sucrose (Fig. S4e–f).

### Increased oligomannose and reduced sialylation enhance the affinity for liver glycoprotein receptors

**Fig. 4:**
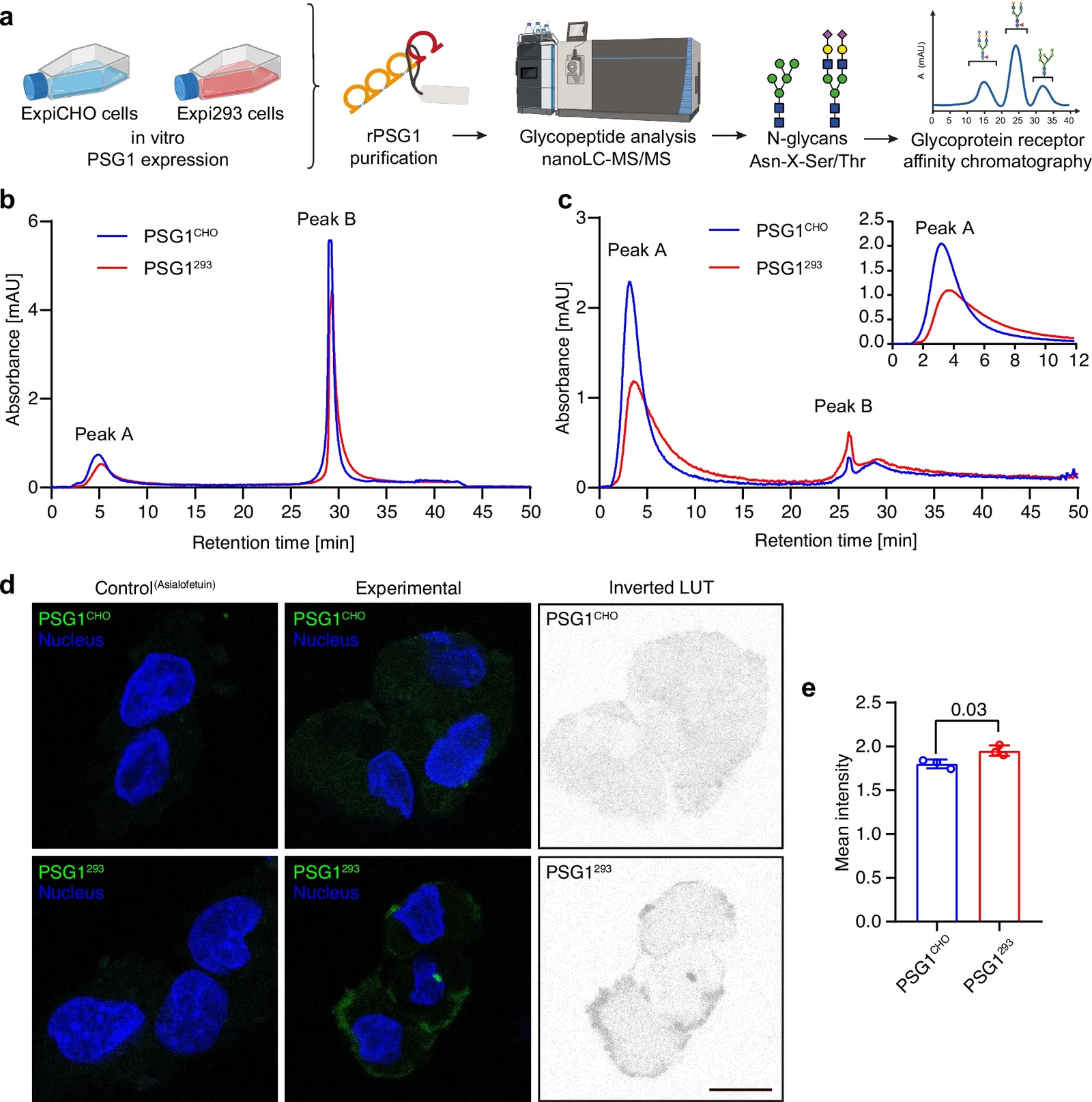
Differential glycosylation of PSG1 modulates interaction with ManR and ASGR. **a** Experimental set-up to be able to use recombinant PSG1 for the affinity chromatography analysis. **b** and **c** Elution of recombinant PSG1-V5His made in CHO and 293 cells following affinity chromatography with immobilized ManR (**b**) or ASGR (**c**). Peak A shows the flow-through peak, and Peak B refers to the elution peak. Chromatograms show the mean of the duplicate measurements for ManR. For the ASGR, a single injection is shown, with the mean flow-through peak of the duplicate measurements shown in the inset (top right). The single injections, in duplicate, and controls for both receptors are shown in Fig. S5a–f. **d** Representative confocal microscopy images per condition, including asialofetuin controls. Scale bar = 10 µm. All images were acquired with identical zoom and gain and matched pixel size. Brightness was enhanced uniformly across all images. **e** Quantitative analysis of Alexa Fluor 488 intensity inside the cytoplasm, averaged per cell and shown per condition (Welch’s t-test; mean ± SD). Abbreviations: ManR: mannose receptor; ASGR: asialoglycoprotein receptor. Partially created in BioRender. Harms, C. (2026) https://BioRender.com/v4ygiwx

Protein clearance by the liver is one of the biological processes that regulates the half-life of proteins in circulation [35]. Therefore, we tested whether differences in PSG1s glycosylation influence the interaction with the two major liver receptors responsible for glycoprotein clearance [35–37]. Due to the limited availability of isolated native PSG1, this experiment was performed using recombinant PSG1 expressed in the two different cell lines, for which we analysed the glycosylation patterns (Fig. 4a). Both recombinant PSG1s bound to the mannose receptor (ManR) column but showed different elution profiles (Fig. 4b, Fig. S5a–b). PSG1^CHO^ eluted first, followed by PSG1^293^, indicating a higher affinity of PSG1^293^ towards ManR compared to PSG1^CHO^ (Peak B). The shift in retention time was also confirmed in the flow-through (Peak A). Additionally, both samples appeared to have different peak shapes, with a more pronounced tailing for PSG1^293^, suggesting a higher abundance of species with increased interaction under the same peak. The same trend was detected when analysing the interaction with the asialoglycoprotein receptor (ASGR). PSG1^293^ had a higher affinity towards ASGR compared to PSG1^CHO^, shown by a pronounced asymmetry and tailing of the flow-through (Peak A) for PSG1^293^ (Fig. 4c, Fig. S5c–d). Immunoblot analysis revealed the presence of PSG1 in the eluted fractions (Fig. S6a), and the fractionation profile is shown in Fig. S6b–c. Incubation of fluorescently labelled recombinant PSG1 with ASGR-expressing HepG2 cells supported this finding: mean cytoplasmic fluorescence intensity was significantly higher after incubation with PSG1^293^ than with PSG1^CHO^ (Welch’s t-test, p = 0.03) (Fig. 4d–e). Co-incubation with the competing ligand asialofetuin reduced the mean fluorescence intensity by 5.2 to 5.9-fold under both conditions, further supporting the involvement of ASGR in PSG1^293^ uptake (Fig. S7). Serum PSG1 levels were significantly reduced in women with early-onset PE in the Hungarian cohort. In contrast, no significant differences were observed between PE patients and controls in the German cohort. In the US cohort, which included only one individual per group, PSG1 levels were lower in the early-onset PE patient compared to the healthy control (Table 1).

## Discussion

We conducted a site-specific glycosylation analysis of PSG1 and report, for the first time, the presence of *O-*glycosylation in this protein. Both PSG1^CTRL^ and PSG1^PE^ carried a core 1 *O-*glycan (T-antigen) in the N domain. At the gestational age studied (third trimester), we detected no difference in *O*-glycosylation between PSG1 purified from pooled control and PE serum. In addition, we identified *N-*glycopeptides at six of the seven *N-*glycosylation sites in both PSG1^CTRL^ and PSG1^PE^. The lack of glycopeptides mapping to the A1 domain (N199) likely explains the lack of glycan-dependent interaction of gal-1 or gal-9 with this domain [9, 10]. While previous MALDI-MS analysis of permethylated released glycans reported that PSG1 complex N-glycans predominantly carry α2,3-linked sialic acids [9], the present LC–MS/MS analysis of underivatized glycopeptides showed a lower sialic acid content, consistent with the known under-representation of sialylated glycans in underivatized positive-ion-mode workflows [38–40]. Interestingly, PSG1 from PE patients showed a higher percentage of oligomannose glycans and reduced relative abundance of complex glycans and terminal sialic acid compared to PSG1 purified from healthy pregnant controls. Additionally, we observed a reduction of fucosylated glycans and an increase in paucimannose structures in the glycopeptides from PSG1^PE^. These results agree with prior studies, which reported an increase in oligomannose [41, 42] and paucimannose structures and a reduction in fucosylation and sialylation in placental proteins of PE patients [41]. This is further supported by observations that ER stress, as occurs in PE, shifts the glycosylation profile of secreted proteins towards oligomannose structures and away from sialylated glycans [43, 44].

Our in vitro ER stress analysis supported this finding, as one of the major α2,3-sialyltransferases (ST3GAL6) expressed by STBs was significantly downregulated after thapsigargin treatment. We furthermore described the downregulation of MAN1C1, a Golgi-resident α1,2-mannosidase involved in the trimming of oligomannose glycans, generating the precursor molecule for complex glycan maturation. This downregulation upon ER stress may lead to the accumulation of oligomannose structures [45]. Similar trends in ST3GAL6 and MAN1C1 expression have been observed in thapsigargin-treated BeWo cells [43]. The normoxic conditions utilized during the experiments mimic the oxygen environment of later pregnancy, aligning with our third-trimester PSG1 analysis.

Immature STBs exhibit increased ER stress markers compared to mature STBs, according to the previously published snRNA sequencing data [30]. Although all STB stages contribute to circulating PSG1, STBim shows the highest expression, followed by STB1. While total mRNA levels of *PSG1* increased in early-onset PE placentas compared to controls, the significant downregulation of *PSG1* in STB1 suggests the pool in early-onset PE is primarily STBim-derived. This shift in origin likely alters the PTMs of PSG1, as STBim expresses higher ER stress markers and lower levels of most glycosyltransferases compared to STB1 and STB2, potentially leading to differential glycosylation patterns observed in the PSG1 isolated from the serum of PE versus control pregnant women. Defective trophoblast differentiation has been described in PE, with trophoblast cells exhibiting altered proinflammatory gene expression indicative of dysregulated differentiation [46]. In the same study, vestigial-like family member 3, a cofactor in the Hippo pathway, showed a strong correlation with PSGs, suggesting that syncytiotrophoblast cells retain canonical markers but exist within a pathological transcriptional context.

Despite these changes in *N*-glycosylation, we did not observe a significant difference in the affinity of gal-1 towards PSG1^PE^ compared to PSG1^CTRL^. Our prior observations using an ELISA-based assay with recombinant PSG1 expressed in CHO and 293 cells, showing that both proteins bind to gal-1, agree with the results presented here [9]. We and others have observed a reduction of PSG1 serum concentration in PE [6, 11–14], potentially impacting the gal-1 that could be protected from inactivation by oxidation [9]. Consistent with our findings on PSG1, reduced serum levels of PSG5 have been reported in early-onset PE compared to healthy controls, accompanied by altered *N-*glycosylation characterized by a shift towards less complex glycan structures and an increase in terminal GlcNAc. Furthermore, placental expression of sialyltransferases was decreased in early-onset PE, leading to a reduction of glycans with terminal sialylation [43].

**Fig. 5:**
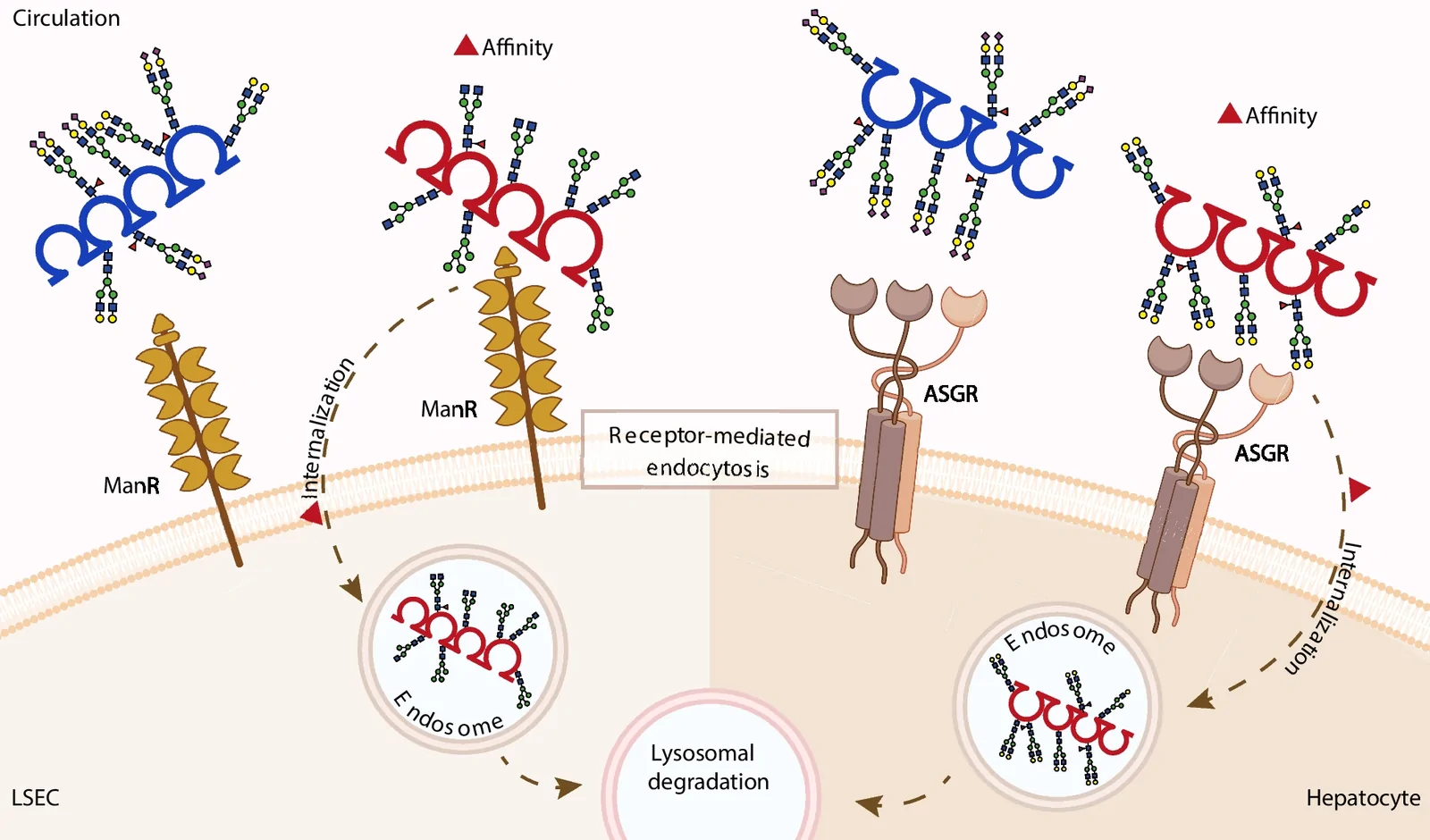
Hypothetical scenario of how altered glycosylation patterns on PSG1 may increase hepatic glycoprotein receptor affinity, potentially contributing to reduced circulating PSG1 levels. Left: enhanced terminal mannose exposure promotes binding to the ManR on LSEC, facilitating PSG1 clearance. Right: increased terminal sialylation reduces recognition by the ASGR on hepatocytes, thereby maintaining PSG1 in circulation. For simplicity, the figure depicts reduced glycan diversity, focusing only on key structures responsible for receptor affinity. Abbreviations: ManR: mannose receptor; ASGR: asialoglycoprotein receptor; LSEC: liver sinusoidal endothelial cells. Partially created in BioRender. Harms, C. (2026) https://BioRender.com/v4ygiwx

Glycoprotein clearance by hepatic receptors, including the ASGR and ManR, is mediated by surface carbohydrates. Terminal sialic acid inhibits the ASGR–glycoprotein ligand interaction and prolongs time in circulation [35]. Clearance of *N-*acetylglucosamine- and α-mannosyl-terminating glycans is mediated by the ManR [36, 37]. Because of the limitations of recruiting and isolating native PSG1 from the sera of pregnant women with PE, binding studies with the receptors that could potentially clear PSG1 from the maternal circulation were performed with recombinant PSG1. We found that PSG1^293^, which had a higher abundance of oligomannose and a lower abundance of sialylated *N*-glycans, bound with higher affinity to both ASGR and ManR than PSG1^CHO^. Given their low abundance, changes in paucimannose glycans were considered unlikely to affect this analysis. Under the same experimental conditions, we previously showed that even slight alterations in antibody–ManR interaction, indicated by increased peak tailing in the flow-through, influenced both uptake by ManR-expressing cells in vitro and circulating concentrations in vivo [47]. This demonstrates that even if PSG1 has a very heterogeneous glycosylation pattern, a switch towards higher levels of oligomannose glycans would likely increase the interaction with the ManR, leading to enhanced clearance rates. In addition, uptake of fluorescently labelled recombinant PSG1 by HepG2 cells, which abundantly express ASGR, was enhanced after incubation with PSG1^293^ compared with PSG1^CHO^. These findings suggest that the changes in *N-*linked glycosylation of PSG1 observed in PE could accelerate its clearance from circulation, thereby explaining the lower serum levels (Fig. 5). This interpretation is supported by studies of placental gene expression in early-onset PE, which reported no significant differences in *PSG* transcript levels compared to healthy controls [17, 48]. These observations further imply that the alterations primarily arise at post-transcriptional or translational stages. Prior reports have shown that differences in glycan composition of recombinant murine Psg23 alter its half-life in circulation via ManR- and ASGR-mediated clearance [26]. Accordingly, the glycosylation differences in PSG1 observed here between preeclamptic and normotensive pregnancies may similarly influence its serum concentration.

Further studies will be needed to address whether these observations can be extended to the other members of the human PSG family. Currently, the lack of reagents to purify the native proteins and measure their concentration with specific ELISAs precludes us from performing these experiments. In addition, the isolation of high amounts of PSG1 from the serum of pregnant women remains challenging. A limitation linked to this is the intracohort pooling of the samples, which reduces statistical power. Future studies should therefore focus on increasing the sample size to validate our findings of altered *N*-glycosylation in PSG1 from third-trimester preeclamptic pregnancies. So far, our study has focused only on the third trimester, in which *O*-glycosylation did not show differences between control and PE pregnancies; however, changes may occur at other time points during the disease, which should be explored in future studies.

In summary, we have determined that PSG1 contains an *O-*linked glycan in the N domain. Moreover, alterations in *N-*glycosylation of PSG1 observed in PE patients compared to healthy pregnant controls may arise from increased ER stress and reduced expression in mature STB, resulting in a more immature-like expression profile. These alterations affect its recognition by hepatic clearance receptors, offering a mechanistic link to the reduced PSG1 levels reported in early-onset PE and highlighting a potential pathway contributing to disease pathophysiology.

## Materials and methods

### Samples and clinical characterization

Native PSG1 was obtained from healthy pregnancies (PSG1^CTRL^) and those complicated with early-onset PE (PSG1^PE^) during the third trimester of pregnancy. Pregnancies were dated according to ultrasound scans between 8–12 weeks of gestation. Patients with multiple pregnancies or fetuses having congenital or chromosomal abnormalities were excluded. Serum samples were collected from three cohorts from three countries (Hungary, Germany, USA), and clinical data were obtained by the corresponding facilities, namely the HUN-REN Research Centre for Natural Sciences of the Hungarian Academy of Sciences, the University of Duisburg-Essen, and the Yale School of Medicine Biobank, and stored at −80 °C until further use. Clinical characteristics of the cohorts, consisting of early-onset PE patients with matched controls classified according to ISSHP guidelines [49], are shown in Table 1.

### Native protein purification and recombinant protein production

PSG1 was purified from (pooled) serum of pregnant women with anti-PSG mAb #4 as previously described [9]. Recombinant PSG1 was expressed in ExpiCHO (PSG1^CHO^) or Expi293 (PSG1^293^) cells. Protein production was described previously [8]. Briefly, PSG1-V5His was purified by affinity chromatography on an anti-PSG mAb#4 column [50].

### Site-specific glycosylation analysis by LC–MS/MS

The *O*- and *N-*glycosylation on native PSG1 from healthy and preeclamptic pregnancies and of recombinant PSG1-V5His expressed in ExpiCHO and Expi293 cells was analysed with nanoLC–MS/MS. Each sample was digested separately with chymotrypsin and trypsin. PSG1 (10 μg) was diluted in 0.1 M triethylammonium bicarbonate buffer with 1% sodium deoxycholate and denatured at 95 °C for 5 min. Disulfide bonds were reduced with 10 mM dithiothreitol for 30 min at 60 °C, followed by alkylation with 20 mM iodoacetamide for 30 min at 37 °C. Samples were brought up to 70% acetonitrile (ACN) and 1 μL of carboxylate-modified magnetic beads (#65,152,105,050,250, Cytiva, Marlborough, MA, USA) was added and incubated for 18 min. The supernatant was discarded, and beads were washed with ACN and 70% ethanol. Digestion was performed by resuspending the beads in 50 mM ammonium bicarbonate, adding 0.5 μL of 0.5 μg/mL trypsin (V5111, Promega, Madison, WI, USA) or chymotrypsin (V1062, Promega) overnight at 37 °C. Afterward, samples were adjusted to 95% ACN. The supernatant was discarded, beads were washed with ACN, and peptides were eluted with 2% dimethyl sulfoxide and 1% formic acid in water. Eluted peptides were dried in a vacuum centrifuge and resuspended in 5 μL of 0.1% formic acid. Measurement was performed on a quadrupole-ion trap-orbitrap MS (Orbitrap Fusion, Thermo Fisher Scientific, Waltham, MA, USA) coupled to a nano-UPLC (Dionex Ultimate 3000 UPLC system, Thermo Fisher Scientific). Peptide separation was performed using an 80 min gradient with increasing ACN concentration and online purification and desalting on a peptide trap (180 μm × 20 mm, 100 Å pore size, 5 μm particle size, Symmetry C18, Waters, Milford, MA, USA) and analytical separation on a 25 cm C18 reversed-phase column (75 μm × 200 mm, 130 Å pore size, 1.7 μm particle size, peptide BEH C18, Waters). Peptides were ionized by electrospray ionization and analysed in positive ion mode using data-dependent acquisition. MS1 scans were accumulated for a maximum of 120 ms or until a charge density of 2 × 10^5^ ions, using the Orbitrap mass analyser. Peptides covering a mass range of m/z 400–1300 with charge states of 2+–5+ were fragmented using higher-energy collisional dissociation. The ion trap mass analyser was used for MS2 scans with a lower m/z limit of 120 and accumulation for a maximum of 60 ms or until a charge density of 1 × 10^5^ ions.

### *O-*glycopeptide analysis

Identification of *O-*glycosylated peptides was performed using the glyco-O-HCD workflow within the open-source software FragPipe with integrated MSFragger-glyco v21.1 [51]. To this end, data obtained after tryptic and chymotryptic digests were searched against a reviewed human protein database obtained from UniProt [52]. MSFragger parameters were set to trypsin or chymotrypsin protein digestion, allowing for two missed cleavages, and peptide length between 5–50 amino acids. Carbamidomethylation was included as fixed modification on cysteine and the protein N-terminal conversion of glutamine or glutamic acid residues to pyro-glutamic acid and oxidation of methionine were included as variable modifications. Data output was checked manually for potentially false positives within peptides carrying an *N-*glycosylation site and results were filtered for a mass deviation of less than 5 ppm.

### *N*-glycopeptide analysis

*N-*glycopeptide analysis was conducted using the open-source software FragPipe with integrated MSFragger-glyco v21.1 [51, 53]. Default workflow for label-free quantification of *N-*glycopeptides (glyco-N-LFQ) was used, and a reviewed human protein database was obtained from UniProt [52]. MSFragger parameters were set to chymotrypsin or trypsin digestion, allowing for two missed cleavages, and peptide length between 5–50 amino acids. Identified glycopeptides were assigned to the corresponding asparagine residues and classified into glycan categories, including complex/hybrid, oligomannose, or paucimannose. Relative glycopeptide abundance was calculated by dividing the intensity of each glycopeptide by the sum of all identified glycopeptide intensities, site-specific and throughout the full-length protein. Analyses were performed on trypsin and chymotrypsin digests, respectively, and results were subsequently combined and averaged to achieve high coverage. Site occupancy was calculated as the sum of the relative abundances of glycopeptides assigned to a given site, divided by the total relative abundance of glycopeptides and corresponding non-glycosylated peptides at that site. Calculations were performed independently for trypsin and chymotrypsin digests, and the resulting values were averaged to obtain the final site occupancy.

### Sialic acid linkage analysis

To further investigate the glycan structures, peptides were incubated with Sialidase S (P0743, New England Biolabs, Ipswich, MA, USA) to discriminate between sialic acid linkages. For the digestion, tryptic and chymotryptic peptides were dried in a vacuum centrifuge and resuspended in glycobuffer supplied by the vendor. After adding Sialidase S, the samples were incubated at 37 °C overnight. The following day, enzyme inactivation was performed by heating at 65 °C for 10 min. The samples were dried in a vacuum centrifuge and resuspended in 0.1% trifluoroacetic acid in water for further desalting by solid-phase extraction with OMIX C18 pipette tips (Agilent, Santa Clara, CA, USA) following the protocol supplied by the vendor. After desalting, the samples were dried in a vacuum centrifuge and resuspended in 0.1% formic acid in water. Measurement and glycopeptide identification were carried out as described above. The results were screened for glycopeptides carrying a sialic acid residue.

### Lectin western blot analysis

Purified native PSG1 was separated by gel electrophoresis (NuPAGE 4%–12% Bis–Tris gels, Thermo Fisher Scientific) in NuPAGE SDS running buffer (Thermo Fisher Scientific) supplemented with 500 μL of NuPAGE Antioxidant (Thermo Fisher Scientific). Subsequently, proteins were transferred to PVDF membranes (Cytiva) using a semi-dry transfer system with transfer buffer containing 1 × NuPAGE transfer buffer (Thermo Fisher Scientific), 1% methanol, and 0.01% NuPAGE Antioxidant. The membrane was blocked with 5% milk powder (#145.3, ROTH, Karlsruhe, Germany) in PBS containing 0.1% Tween 20 after which it was incubated overnight at 4 °C with lectin PNA at 20 μg/mL (BA-2301–5, EY Laboratories, San Mateo, CA, USA). The following day, membranes were washed and incubated for 1 h at room temperature with HRP-conjugated streptavidin at 1/10000 (#189,733, Millipore, Billerica, MA, USA). After additional washes, signals were developed using a chemiluminescent detection system (Amersham Imager 600, GE, Cytiva) with an enhanced chemiluminescent substrate (#NEL104001EA, Perkin Elmer, Waltham, MA, USA). Quantification was performed using ImageJ software (version 1.53), normalizing lectin signals to total PSG1. An additional western blot was performed to control the specificity of PNA. For this purpose, fetuin (F3004, Sigma-Aldrich, St. Louis, Missouri, USA) was digested overnight with neuraminidase A (P0722S, New England Biolabs) or mock-digested with water, in the buffer provided by the manufacturer and following their instructions. Gel electrophoresis and blotting were performed as described above, loading 0.5 and 1 µg each of fetuin, asialofetuin (A4781, Sigma-Aldrich), the neuraminidase A-digested fetuin, and the mock-digested fetuin, alongside 1 µL neuraminidase A alone.

### Public dataset analysis: single-nucleus RNA sequencing

PSG1 expression was analysed in a previously published snRNA sequencing dataset, consisting of 6 term controls and 6 early-onset PE placental samples [30]. In this dataset, gestational age effects were accounted for during both data integration and differential expression analysis. Gestational week was incorporated as a covariate during integration, while a cell-type-specific preterm score derived from independent datasets was included in the differential expression model to adjust for gestational age-associated transcriptional variation. Previously processed data were used to visualize the expression of genes of interest.

### Public dataset analysis: bulk placental RNA sequencing

Publicly available placental gene expression data were analysed as part of a previously published combined dataset including controls and matched preeclampsia samples. This dataset comprised 330 placental samples (173 controls and 157 PE), generated from an institutional BioBank cohort and seven independent microarray studies, including GSE30186, GSE10588, GSE24129, GSE25906, GSE43942, GSE4707, GSE44711, and GSE75010 [54]. To enable analyses focussing on early-onset PE and accounting for gestational age, the dataset was restricted to studies with available metadata. This filtering step reduced the dataset to two constituent studies, including the BioBank cohort (n = 142, control = 85, PE = 57), which were retained for downstream analyses. Gene expression of PSG1 was analysed using a linear regression. Expression levels were modelled as a function of PE status while accounting for the dataset of origin and gestational age.

### Label-free molecular interaction analysis of PSG1 and gal-1

Molecular interaction analysis was performed by interferometry on a Creoptix wave system (Malvern Panalytical, Malvern, Worcestershire, UK) adjusted based on a protocol described elsewhere [9]. Briefly, recombinant and native PSG1 from the German cohort in 10 mM sodium acetate, pH 4.0, was immobilized on a 4PCH WAVE chip (Creoptix, Malvern Panalytical) using standard amine coupling, following the vendor’s instructions. A control surface without immobilized protein was used for background correction. The flow rate was set to 100 μL/min at 25 °C and HBS-EP (0.01 M HEPES pH 7.4, 0.15 M NaCl, 3 mM EDTA, and 0.005% Surfactant P20) was used as running buffer. For multiple cycle kinetics, recombinant human gal-1 (#45,039, PeproTech, Thermo Fisher Scientific) at concentrations ranging from 3 μM to 117 nM was injected for 200 s, followed by a 1000 s dissociation period. To regenerate the surface after each gal-1 injection, a solution containing 1 M NaCl and 20 mM NaOH in HBS-EP buffer was injected for 30 s. Competition experiments were performed with 5 μM gal-1 by adding sucrose or lactose at 1.43 mM to the buffer 30 min before injection. Analysis was performed using the WAVEcontrol software (v4.2.0) with a 1:1 kinetic fit.

### Mannose and asialoglycoprotein receptor affinity chromatography

The full extracellular domain of human ManR C-type 1 with a C-terminal histidine tag and AviTag, was transiently expressed in HEK-F cells and purified using Ni–NTA affinity chromatography and size-exclusion chromatography [55]. The ASGR1 domain of the human ASGR, containing a N-terminal histidine tag and AviTag (GS1-H82Q3), was purchased from AcroBiosystems (Newark, DE, USA). Affinity columns for each receptor were prepared using 1 mL Streptavidin Sepharose High-Performance resin (Cytiva), loaded with either 6 mg of ManR or 400 μg of ASGR1. The receptor-coupled beads were packed into 5/50 Tricorn columns (Cytiva) and operated on an ÄKTA go system (Cytiva) at room temperature, with data collection and device control managed by Unicorn software (version 7.5, Cytiva). For ManR two affinity columns were connected in tandem. After column equilibration (12 mM Tris, 140 mM NaCl, 2.5 mM CaCl₂, pH 7.4), 15 μg of PSG1-V5His made in ExpiCHO or made in Expi293 cells were injected. A 24 min gradient from 0%–100% mobile phase B (12 mM Tris, 140 mM NaCl, 2 mM EDTA, pH 7.4) was applied, followed by a 12 min hold at 100% mobile phase B. Re-equilibration with mobile phase A was performed after each run. The flow rate was maintained at 0.50 mL/min, and absorbance at 280 nm was detected. The experiments were conducted in duplicate for ManR, with data represented as mean, and as a single measurement for ASGR1. Following affinity chromatography with immobilized ManR, four elution fractions were collected and analysed by western blot with the complete fraction loaded in a single lane.

### Quantification of PSG1 by ELISA

The concentration of PSG1 was obtained using the PSG1 ELISA Kit (DY6799-05, R&D Systems) following the manufacturer’s recommendations. Serum samples were diluted as needed to obtain values within the PSG1 standards provided in the kit (1/1 000; 1/5 000, or 1/10 000).

### mRNA expression analysis of ER-stressed syncytiotrophoblasts in vitro

Primary cytotrophoblast cells from human term placenta (#1230, The Amnion Foundation, Winston-Salem, NC, USA) were seeded at 50 000 cells/cm^2^ on a 6-well plate, pre-coated with Matrigel (1% for 1 h at 37 °C), under CTBPRO2GRO media (1234, The Amnion Foundation, Winston-Salem, NC, USA). After 24 h, cells were differentiated to STBs with differentiation basal media (DMEM/F12; 0.1 mM 2-mercaptoethanol, 0.5% penicillin/streptomycin, 0.3% BSA, 1% ITS-X supplement, 2.5 μM Y27632, 4% knockout serum replacement) supplemented with 25 μM Forskolin for 6 days in the incubator at 21% O_2_ and 5% CO_2_. Following this time, cells were exposed to differentiation basal media supplemented with DMSO (0.001%) or 0.1 μM thapsigargin for 24 h. Then, cells were resuspended in TRIzol (R2050-1, Zymo Research, Irvine, CA, USA) and total RNA was purified using the PureLink RNA Mini Kit (#12183018A, Invitrogen, Carlsbad, CA, USA). RNA concentration was determined using a NanoDrop One (Thermo Fisher Scientific) and the iScript Advanced cDNA Synthesis Kit (#1725038, Bio-Rad Laboratories, Hercules, CA, USA) was used to convert RNA into cDNA following the manufacturer's recommendations. Quantitative real-time PCR (RT-qPCR) was performed using the QuantStudio 5 (Thermo Fisher Scientific) with *Power* SYBR™ Green PCR Master Mix (#4367659, Thermo Fisher Scientific). For each primer pair, a negative control without cDNA was included. mRNA expression was normalized to GAPDH and YWHAZ. All primer sequences are listed in Table S2. Differences between groups were assessed using multiple t-tests with Benjamini–Hochberg correction.

### Cell uptake

HepG2 cells (ACC 180, DSMZ, Braunschweig, Germany) were seeded at 10 000 cells per well in Millicell EZ slide 8-well glass slides (PEZGS0896, Millipore) and cultured overnight in RPMI (#11875093, Gibco™, Waltham, MA, USA) with 10% FCS at 37 °C and 5% CO_2_. Recombinant PSG1 was labelled using the AlexaFluor 488 Protein labelling kit (A10235, Thermo Fisher Scientific) and added to the cells at a final concentration of 5 µg/mL in RPMI. Cells were incubated with the labelled protein at 37 °C and 5% CO_2_ for 80 min. For specificity controls, labelled protein was co-incubated with 200 µg/mL asialofetuin (A4781, Sigma-Aldrich), a high-affinity ligand of ASGR [56]. After incubation, cells were washed with ice-cold PBS. Surface-bound protein was removed with glycine–HCl buffer (0.2 M, pH 3, 3 min on ice), followed by further washes with PBS. Cells were fixed with 4% formaldehyde for 10 min at room temperature. Nuclei were stained with DAPI (0.5 µg/mL, D8417, Sigma-Aldrich) for 20 min, and slides were mounted in Prolong mounting medium (P36934, Invitrogen). Images were acquired with an inverted Leica TCS SP8 X microscope (Leica-Microsystems, Wetzlar, Germany) using a 63 × oil-immersion objective. Alexa-Fluor-labelled PSG1^CHO^ or PSG1^293^ and DAPI were excited at 488 and 405 nm, respectively, with emission collected using HyD detectors. Images were acquired using LAS X software. Confocal images were analysed in QuPath (v. 0.7.0- × 64). Cells were segmented on the DAPI channel, and mean fluorescence intensity of the 488 channel within the cytoplasm was measured. Per-cell values were averaged across images and replicates (n = 3, 4 images each). Total cell counts included in the analysis are shown in Table S3. Conditions were compared using an unpaired Welch’s t-test.

## Supplementary Information

Supplementary Material 1.

## Data Availability

Mass spectrometry data are deposited in the public data repository glycoPOST and are available under the accession number GPST000748 [57]. All other data are available from the corresponding author upon request.

## References

[1] Tóth E, Györffy D, Posta M, Hupuczi P, Balogh A, Szalai G, et al. Decreased expression of placental proteins in recurrent pregnancy loss: functional relevance and diagnostic value. Int J Mol Sci. 2024;25(3):1865. 10.3390/IJMS25031865. PMC10855863 38339143

[2] Bohn H. Nachweis und Charakterisierung von Schwangerschaftsproteinen in der menschlichen Placenta, sowie ihre quantitative immunologische Bestimmung im Serum schwangerer Frauen. Arch Gynakol. 1971;210(4):440–57. 10.1007/BF01628222. 5001318

[3] Towler CM, Horne CHW, Jandial V, Campbell DM, MacGillivray I. Plasma levels of pregnancy-specific β1-glycoprotein in normal pregnancy. Br J Obstet Gynaecol. 1976;83(10):775–9. 10.1111/j.1471-0528.1976.tb00743.x. 990216

[4] Lee JN, Grudzinskas JG, Chard T. Circulating levels of pregnancy proteins in early and late pregnancy in relation to placental tissue concentration. Br J Obstet Gynaecol. 1979;86(11):888–90. 10.1111/J.1471-0528.1979.TB10717.X. 315792

[5] Zhou GQ, Baranov V, Zimmermann W, Grunert F, Erhard B, Mincheva-Nilsson L, et al. Highly specific monoclonal antibody demonstrates that pregnancy-specific glycoprotein (PSG) is limited to syncytiotrophoblast in human early and term placenta. Placenta. 1997;18(7):491–501. 10.1016/0143-4004(77)90002-9. 9290143

[6] Rattila S, Dunk CE, Im M, Grichenko O, Zhou Y, Cohen M, et al. Interaction of Pregnancy-Specific Glycoprotein 1 With Integrin α5β1 Is a Modulator of Extravillous Trophoblast Functions. Cells. 2019;8(11):1369. 10.3390/cells8111369. PMC6912793 31683744

[7] Blois SM, Sulkowski G, Tirado-González I, Warren J, Freitag N, Klapp BF, et al. Pregnancy-specific glycoprotein 1 (PSG1) activates TGF-β and prevents dextran sodium sulfate (DSS)-induced colitis in mice. Mucosal Immunol. 2014;7(2):348–58. 10.1038/mi.2013.53. PMC3844031 23945545

[8] Rattila S, Kleefeldt F, Ballesteros A, Beltrame JS, Ribeiro ML, Ergün S, et al. Pro-angiogenic effects of pregnancy-specific glycoproteins in endothelial and extravillous trophoblast cells. Reproduction. 2020;160(5):737–50. 10.1530/REP-20-0169. PMC7575825 33065549

[9] Mendoza M, Lu D, Ballesteros A, Blois SM, Abernathy K, Feng C, et al. Glycan characterization of pregnancy-specific glycoprotein 1 and its identification as a novel Galectin-1 ligand. Glycobiology. 2020;30(11):895–909. 10.1093/glycob/cwaa034. PMC7581653 32280962

[10] Mendoza M, Ballesteros A, Rendon-Correa E, Tonk R, Warren J, Snow AL, et al. Modulation of galectin-9 mediated responses in monocytes and T-cells by pregnancy-specific glycoprotein 1. J Biol Chem. 2024;300(9):107638. 10.1016/j.jbc.2024.107638. PMC11403483 39121996

[11] Tu C, Tao F, Qin Y, Wu M, Cheng J, Xie M, et al. Serum proteins differentially expressed in early- and late-onset preeclampsia assessed using iTRAQ proteomics and bioinformatics analyses. PeerJ. 2020;8:e9753. 10.7717/PEERJ.9753. PMC7473043 32953262

[12] Temur M, Serpim G, Tuzluoğlu S, Taşgöz FN, Şahin E, Üstünyurt E. Comparison of serum human pregnancy-specific beta-1-glycoprotein 1 levels in pregnant women with or without preeclampsia. J Obstet Gynaecol. 2020;40(8):1074–8. 10.1080/01443615.2019.1679734. 31790616

[13] Wang Q, Fang Y, Li Y, Liu H, Zhu M, Hu X, et al. Pregnancy-specific beta-1-glycoprotein 1 increases HTR-8/SVneo cell migration through the Orai1/Akt signaling pathway. Biomolecules. 2024;14(3):293. 10.3390/biom14030293. PMC10968057 38540713

[14] Grudzinskas JG, Gordon YB, Menabawey M, Lee JN, Wadsworth J, Chard T. Identification of high-risk pregnancy by the routine measurement of pregnancy-specific β1-glycoprotein. Am J Obstet Gynecol. 1983;147(1):10–2. 10.1016/0002-9378(83)90075-3. 6604456

[15] Poon LC, Shennan A, Hyett JA, Kapur A, Hadar E, Divakar H, et al. The International Federation of Gynecology and Obstetrics (FIGO) initiative on pre-eclampsia: a pragmatic guide for first-trimester screening and prevention. Int J Gynaecol Obstet. 2019;145(S1):1–33. 10.1002/IJGO.12802. PMC6944283 31111484

[16] Burton GJ, Jauniaux E. Placental Oxidative Stress: From Miscarriage to Preeclampsia. J Soc Gynecol Investig. 2004;11(6):342–52. 10.1016/j.jsgi.2004.03.003. 15350246

[17] Than NG, Romero R, Tarca AL, Kekesi KA, Xu Y, Xu Z, et al. Integrated systems biology approach identifies novel maternal and placental pathways of preeclampsia. Front Immunol. 2018;9:1661. 10.3389/FIMMU.2018.01661. PMC6092567 30135684

[18] Szabo S, Mody M, Romero R, Xu Y, Karaszi K, Mihalik N, et al. Activation of villous trophoblastic p38 and ERK1/2 signaling pathways in preterm preeclampsia and HELLP syndrome. Pathol Oncol Res. 2015;21(3):659–68. 10.1007/S12253-014-9872-9. PMC5987200 25583406

[19] Castro KR, Prado KM, Lorenzon AR, Hoshida MS, Alves EA, Francisco RPV, et al. Serum from preeclamptic women triggers endoplasmic reticulum stress pathway and expression of angiogenic factors in trophoblast cells. Front Physiol. 2022;12:799653. 10.3389/FPHYS.2021.799653. PMC8855099 35185601

[20] Burton GJ, Yung HW. Endoplasmic reticulum stress in the pathogenesis of early-onset pre-eclampsia. Pregnancy Hypertens. 2011;1(1):72–8. 10.1016/j.preghy.2010.12.002. PMC3252240 22242213

[21] Kirkgöz K, Vogtmann R, Xie Y, Zhao F, Riedel A, Adam LM, et al. Placental glycosylation senses the anti-angiogenic milieu induced by human sFLT1 during pregnancy. J Reprod Immunol. 2024;164:104284. 10.1016/J.JRI.2024.104284. 38908337

[22] Sukhikh GT, Ziganshina MM, Nizyaeva NV, Kulikova GV, Volkova JS, Yarotskaya EL, et al. Differences of glycocalyx composition in the structural elements of placenta in preeclampsia. Placenta. 2016;43:69–76. 10.1016/J.PLACENTA.2016.05.002. 27324102

[23] Minas V, Mylonas I, Schiessl B, Mayr D, Schulze S, Friese K, et al. Expression of the blood-group-related antigens Sialyl Lewis a, Sialyl Lewis x and Lewis y in term placentas of normal, preeclampsia, IUGR- and HELLP-complicated pregnancies. Histochem Cell Biol. 2007;128(1):55–63. 10.1007/S00418-007-0293-7. 17551747

[24] Wang H, Shi Y, Ma J, Wang W, Gao J, Zhao L, et al. Integrated proteomic and N-glycoproteomic profiling of placental tissues of patients with preeclampsia. Int J Womens Health. 2023;15:59–68. 10.2147/IJWH.S387672. PMC9844819 36660462

[25] Gómez-Gutiérrez AM, Parra-Sosa BE, Bueno-Sánchez JC. Glycosylation Profile of the Transferrin Receptor in Gestational Iron Deficiency and Early-Onset Severe Preeclampsia. J Pregnancy. 2019;2019:9514546. 10.1155/2019/9514546. PMC6378037 30854239

[26] Mi Y, Lin A, Fiete D, Steirer L, Baenziger JU. Modulation of mannose and asialoglycoprotein receptor expression determines glycoprotein hormone half-life at critical points in the reproductive cycle. J Biol Chem. 2014;289(17):12157–67. 10.1074/JBC.M113.544973. PMC4002119 24619407

[27] Bueno-Sánchez JC, Gómez-Gutiérrez AM, Maldonado-Estrada JG, Quintana-Castillo JC. Expression of placental glycans and its role in regulating peripheral blood NK cells during preeclampsia: a perspective. Front Endocrinol (Lausanne). 2023;14:1087845. 10.3389/fendo.2023.1087845. PMC10190602 37206444

[28] Dimitriadou F, Phocas I, Mantzavinos T, Sarandakou A, Rizos D, Zourlas PA. Discordant secretion of pregnancy specific beta 1-glycoprotein and human chorionic gonadotropin by human pre-embryos cultured in vitro. Fertil Steril. 1992;57(3):631–6. 10.1016/S0015-0282(16)54912-7. 1740210

[29] Osborne JC, Rosen SW, Nilsson B, Calvert I, Bohn H. Physicochemical Studies of Pregnancy-Specific β1-Glycoprotein: Unusual Ultracentrifugal and Circular Dichroic Properties. Biochemistry. 1982;21(22):5523–8. 10.1021/bi00265a022. 6983365

[30] Nonn O, Debnath O, Valdes DS, Sallinger K, Secener AK, Fischer C, et al. Senescent syncytiotrophoblast secretion during early onset preeclampsia. Hypertension. 2025;82(5):787–99. 10.1161/HYPERTENSIONAHA.124.23362. PMC12002046 39440423

[31] Lotan R, Skutelsky E, Danon D, Sharon N. The purification, composition, and specificity of the anti-T lectin from peanut (*Arachis hypogaea*). J Biol Chem. 1975;250(21):8518–23. 10.1016/S0021-9258(19)40790-4. 811657

[32] Grant OC, Wentworth D, Holmes SG, Kandel R, Sehnal D, Wang X, et al. Generating 3D models of complex carbohydrates with GLYCAM-Web. Nat Methods. 2026;23(4):720–3. 10.1038/s41592-026-03033-w. 41872371

[33] Varadi M, Bertoni D, Magana P, Paramval U, Pidruchna I, Radhakrishnan M, et al. AlphaFold Protein Structure Database in 2024: providing structure coverage for over 214 million protein sequences. Nucleic Acids Res. 2024;52(D1):D368–75. 10.1093/NAR/GKAD1011. PMC10767828 37933859

[34] Stowell SR, Arthur CM, Mehta P, Slanina KA, Blixt O, Leffler H, et al. Galectin-1, -2, and -3 Exhibit Differential Recognition of Sialylated Glycans and Blood Group Antigens. J Biol Chem. 2008;283(15):10109–23. 10.1074/JBC.M709545200. PMC2442294 18216021

[35] Ashwell G, Morell AG. The Role of Surface Carbohydrates in the Hepatic Recognition and Transport of Circulating Glycoproteins. Adv Enzymol Relat Areas Mol Biol. 1974;41:99–128. 10.1002/9780470122860.ch3. 4609051

[36] Baynes JW, Wold F. Effect of glycosylation on the in vivo circulating half-life of ribonuclease. J Biol Chem. 1976;251(19):6016–24. 10.1016/S0021-9258(17)33053-3. 972151

[37] Stockert RJ, Morell AG, Scheinberg IH. The existence of a second route for the transfer of certain glycoproteins from the circulation into the liver. Biochem Biophys Res Commun. 1976;68(3):988–93. 10.1016/0006-291x(76)91243-2. 1259744

[38] Yang S, Zhang L, Thomas S, Hu Y, Li S, Cipollo J, et al. Modification of sialic acids on solid phase: accurate characterization of protein sialylation. Anal Chem. 2017;89(12):6330–5. 10.1021/ACS.ANALCHEM.7B01048. PMC5583724 28505427

[39] Nishikaze T. Sialic acid derivatization for glycan analysis by mass spectrometry. Proc Jpn Acad Ser B Phys Biol Sci. 2019;95(9):523–37. 10.2183/PJAB.95.036. PMC6856002 31708496

[40] de Haan N, Yang S, Cipollo J, Wuhrer M. Glycomics studies using sialic acid derivatization and mass spectrometry. Nat Rev Chem. 2020;4(5):229–42. 10.1038/S41570-020-0174-3. 37127981

[41] Robajac D, Vanhooren V, Masnikosa R, Miković Ž, Mandić V, Libert C, et al. Preeclampsia transforms membrane N-glycome in human placenta. Exp Mol Pathol. 2016;100(1):26–30. 10.1016/j.yexmp.2015.11.029. 26655437

[42] Campuzano M, Bueno-Sánchez J, Agudelo-Jaramillo B, Quintana-Castillo JC, Chaouat GC, Maldonado-Estrada JG. Glycan expression in chorionic villi from histocultures of women with early-onset preeclampsia: Immunomodulatory effects on peripheral natural killer cells. J Reprod Immunol. 2020;142:103212. 10.1016/j.jri.2020.103212. 33032074

[43] Yung HW, Zhao X, Glover L, Burrin C, Pang PC, Jones CJP, et al. Perturbation of placental protein glycosylation by endoplasmic reticulum stress promotes maladaptation of maternal hepatic glucose metabolism. iScience. 2023;26(1):105911. 10.1016/j.isci.2022.105911. PMC9843443 36660474

[44] Wong MY, Chen K, Antonopoulos A, Kasper BT, Dewal MB, Taylor RJ, et al. XBP1s activation can globally remodel N-glycan structure distribution patterns. Proc Natl Acad Sci U S A. 2018;115(43):E10089–98. 10.1073/pnas.1805425115. PMC6205500 30305426

[45] Ladaigue S, Lefranc AC, Balde K, Quitoco M, Bacquer E, Busso D, et al. A role for endothelial alpha-mannosidase MAN1C1 in radiation-induced immune cell recruitment. iScience. 2022;25(12):105482. 10.1016/J.ISCI.2022.105482. PMC9668741 36404925

[46] Plazyo O, Chopp LB, Peela R, Young K, Zhang H, Bogle R, et al. Defective Trophoblast Differentiation, Endothelial Dysfunction, and Immune Dysregulation in Preeclampsia Coalesce on a Placental VGLL3-Centered Gene Network. Circulation. 2026;153(22):1743–60. 10.1161/CIRCULATIONAHA.125.076218. PMC13120743 41953989

[47] Baumeister J, Meudt M, Wei W, Ebert S, Rosenau F, Mizaikoff B, et al. Selective clearance of monoclonal antibodies via the mannose receptor is dependent on glycan pairing. Sci Rep. 2026;16(1):8489. 10.1038/s41598-026-40268-1. PMC12972077 41796113

[48] Várkonyi T, Nagy B, Füle T, Tarca AL, Karászi K, Schönléber J, et al. Microarray profiling reveals that placental transcriptomes of early-onset HELLP syndrome and preeclampsia are similar. Placenta. 2011;32(Suppl):S21-29. 10.1016/J.PLACENTA.2010.04.014. PMC3917714 20541258

[49] Magee LA, Brown MA, Hall DR, Gupte S, Hennessy A, Karumanchi SA, et al. The 2021 International Society for the Study of Hypertension in Pregnancy classification, diagnosis & management recommendations for international practice. Pregnancy Hypertens. 2022;27:148–69. 10.1016/J.PREGHY.2021.09.008. 35066406

[50] Moore T, Williams JM, Becerra-Rodriguez MA, Dunne M, Kammerer R, Dveksler G. Pregnancy-specific glycoproteins: evolution, expression, functions and disease associations. Reproduction. 2022;163(2):R11-23. 10.1530/REP-21-0390. 35007205

[51] Polasky DA, Yu F, Teo GC, Nesvizhskii AI. Fast and comprehensive N- and O-glycoproteomics analysis with MSFragger-Glyco. Nat Methods. 2020;17(11):1125–32. 10.1038/S41592-020-0967-9. PMC7606558 33020657

[52] Bateman A, Martin MJ, Orchard S, Magrane M, Ahmad S, Alpi E, et al. UniProt: the universal protein knowledgebase in 2023. Nucleic Acids Res. 2023;51(D1):D523–31. 10.1093/NAR/GKAC1052. PMC9825514 36408920

[53] Kong AT, Leprevost FV, Avtonomov DM, Mellacheruvu D, Nesvizhskii AI. MSFragger: ultrafast and comprehensive peptide identification in mass spectrometry–based proteomics. Nat Methods. 2017;14(5):513–20. 10.1038/nmeth.4256. PMC5409104 28394336

[54] Leavey K, Benton SJ, Grynspan D, Kingdom JC, Bainbridge SA, Cox BJ. Unsupervised Placental Gene Expression Profiling Identifies Clinically Relevant Subclasses of Human Preeclampsia. Hypertension. 2016;68(1):137–47. 10.1161/HYPERTENSIONAHA.116.07293. 27160201

[55] Baumeister J, Meudt M, Ebert S, Rosenau F, Mizaikoff B, Blech M, et al. Decoding the mannose receptor-mAb interaction: the importance of high-mannose N-glycans and glycan-pairing. MAbs. 2024;16(1):2400414. 10.1080/19420862.2024.2400414. PMC11385167 39245969

[56] Rodríguez-Castejón J, Fernández-Muro P, Beraza-Millor M, Solinís MÁ, Rodríguez-Gascón A, del Pozo-Rodríguez A. Asialofetuin-coupled lipid-based nanosystems to target the asialoglycoprotein receptor: delivering genes to hepatocytes for the treatment of Fabry disease. Eur J Pharm Sci. 2025;210:107118. 10.1016/J.EJPS.2025.107118. 40328357

[57] Watanabe Y, Aoki-Kinoshita KF, Ishihama Y, Okuda S. GlycoPOST realizes FAIR principles for glycomics mass spectrometry data. Nucleic Acids Res. 2021;49(D1):D1523–8. 10.1093/NAR/GKAA1012. PMC7778884 33174597

