# Supplementary material for: Syncytiotrophoblast differentiation defects drive ER stress and reprogram placental PSG1 glycosylation in preeclampsia
Source: Mol Biomed. 2026 Oct 30;7:235. doi: 10.1186/s43556-026-00634-x (PMC13722672; doi:10.1186/s43556-026-00634-x)
Supplement: Supplementary file 1 [file 43556_2026_634_MOESM1_ESM.docx]

**Supplemental information**

**Syncytiotrophoblast differentiation defects drive ER stress and reprogram placental PSG1 glycosylation in preeclampsia**

Authors: Charlotte Harms, Yiru Wang, James Warren, Julia Baumeister, Fabian Higel, Cristina Antich, Marc Ferrer, Jennifer J. Hill, Mariana G. Garcia, Alexandra Gellhaus, Nándor Gábor Than, Florian Herse, Stefan Verlohren, Gabriela S. Dveksler, Sandra M. Blois

**Supplementary Tables**

**Table S1: N-glycopeptides potentially carrying α2,6-linked sialic acid residues**. Identified N-glycopeptides from tryptic and chymotryptic digests of PSG1 that retained sialic acid residues after sialidase S treatment, as determined by mass spectrometry. Glycopeptide sequences are presented using the one-letter amino acid code.

| **PSG1** | **Observed**  **m/z** | **Theoretical m/z** | **Delta mass [Da]** | **Delta mass [ppm]** | **Charge** | **Residue** | **Domain** | **Glycopeptide** | **Composition % mass** |
| --- | --- | --- | --- | --- | --- | --- | --- | --- | --- |
| PE | 1084.115 | 1084.114 | 0.0018 | 1.6603 | 3 | 109-120 | N | IQNVTREDAGSY | HexNAc(4)Hex(4)Fuc(1)NeuAc(1) % 1897.6821 |

Abbreviations: PE: native PSG1 from preeclamptic pregnant women; m/z: mass-to-charge ratio; HexNAc: *N*-acetylhexosamine; Hex: hexose; Fuc: fucose; NeuAc: *N*-acetylneuraminic acid.

**Table S2: RT-qPCR primer sequences.** List of primer pairs and their sequences used to analyse the *N*-glycan enzyme machinery and to detect ER stress in human primary syncytiotrophoblasts.

| **Primer** | **Forward** | **Reverse** |
| --- | --- | --- |
| *GAPDH* | GAAGGTGAAGGTCGGAGTCAA | GGAAGATGGTGATGGGATTTC |
| *YWHAZ* | CCTGCATGAAGTCTGTAACTGAG | TTGAGACGACCCTCCAAGATG |
| *MAN1A1* | ATTTGGAACCCTGCATTTGG | TGATGTTGACCCCACTGTCC |
| *MAN1C1* | AGCATCTTGGCGGAGTTTG | GCTGAGGAAGTTGGGGTAGA |
| *MGAT1* | CCTATGACCGAGATTTCCTCG | GCTGTCCCTGCCCGTATA |
| *MGAT2* | GGAAGCTGCATTTTGTGTGG | CATTCAGGGCACTCTTGCTG |
| *MGAT3* | GAGTCCAACTTCACGGCTTAT | GATGTACTCGAAGGTGCCATT |
| *B4GALT1* | GTATTTTGGAGGTGTCTCTGCTC | GGGCGAGATATAGACATGCCT |
| *ST3GAL4* | GGGATGTCAATCCTAAACAGA | CTTCTGCTTAATCTTCCGTGG |
| *ST3GAL6* | GAGGTTTCATCAGTTTCACC | AGGGCAAATCAAACTTATCG |
| *ST6GAL1* | GCAAAGATCAGAGTGAAACAG | CACCTCATCGCAGACATG |
| *HSPA5* | TGTTCAACCAATTATCAGCAAACTC | TTCTGCTGTATCCTCTTCACCA |
| *DDIT3* | GGAAACAGAGTGGTCATTCCC | CTGCTTGAGCCGTTCATTCTC |
| *XBP1s* | AACCAGGAGTTAAGACAGCG | CTGCACCCTCTGCGGA |
| *PPP1R15A* | ATGATGGCATGTATGGTGAGC | AACCTTGCAGTGTCCTTATCAG |
| *HERPUD1* | AACGGCATGTTTTGCATCTG | GGGGAAGAAAGGTTCCGAAG |

**Table S3: Cell count for confocal microscopy analysis.** Number of cells included in the quantitative analysis of Alexa Fluor 488 intensity in the cytosol of HepG2 cells.

| **Replicate** | **1** | | | | **2** | | | | **3** | | | | **Total cell count** |
| --- | --- | --- | --- | --- | --- | --- | --- | --- | --- | --- | --- | --- | --- |
| **Image** | **1** | **2** | **3** | **4** | **1** | **2** | **3** | **4** | **1** | **2** | **3** | **4** |  |
| PSG1^CHO^ | 22 | 16 | 16 | 26 | 16 | 29 | 51 | 99 | 30 | 33 | 26 | 40 | 404 |
| PSG1^293^ | 32 | 21 | 20 | 46 | 25 | 11 | 34 | 31 | 65 | 48 | 51 | 48 | 432 |

**Supplementary Figure 1**

**Fig. S1: O-glycopeptide identification in PSG1 by LC-MS/MS. a** Annotated MS/MS spectra of a tryptic O-glycopeptide in the N domain of PSG1^CTRL^ (m/z 982.483) analysed using FragPipe with integrated MSFragger-glyco. **b** Annotated O-glycopeptide MS/MS spectra of a tryptic O-glycopeptide in the N domain of PSG1^PE^ (m/z 982.483) using FragPipe with integrated MSFragger-glyco.

**Supplementary Figure 2**

**Fig. S2: *O*-glycopeptide identification in PSG1 by Western Blot. a** Western blot analysis of native PSG1 to confirm the presence of *O*-glycosylation (mean ± SD). Detection of *O*-glycans was performed using the lectin PNA, which binds to core-1 and -2 *O*-glycans. The anti-PSG antibody BAP3 was used to normalize PSG loading. **b** Western blot to validate the specificity of PNA binding using Fetuin (0.5 µg and 1 µg) as a negative control and Asialofetuin (0.5 µg and 1 µg) and neuraminidase-digested Fetuin (0.5 µg and 1 µg) as positive controls.

**Supplementary Figure 3**

**Fig. S3: *N*-glycosylation pattern of recombinant PSG1 as determined by nanoLC-MS/MS**. **a** Relative abundance of *N*-glycan classes in recombinant PSG1 made in ExpiCHO and Expi293 cells. **b** Visualization of the most abundant *N*-glycan structures with their relative abundance at each *N*-glycosylation site of recombinant PSG1 expressed in ExpiCHO and Expi293 cells.

**Supplementary Figure 4**

**Fig. S4: Label-free interaction kinetics of PSG1 and gal-1 and its inhibition by the addition of lactose.**

**a** – **c** Creoptix WAVE multiple-cycle kinetics experiment showing the interaction of gal-1 with immobilized PSG1: (**a**) PSG1^CTRL^, (**b**) PSG1^PE^, and (**c**) recombinant PSG1^293^. Gal-1 was injected at concentrations ranging from 3 μM to 100 nM. **d** Kinetic constants and affinity of the interaction between native or recombinant PSG1 and gal-1. **e** – **g** Creoptix WAVE competitive binding assay, injecting gal-1 at a concentration of 5 μM with the addition of sucrose or lactose at 1.43 mM as indicated, with immobilized native PSG1^CTRL^ (**e**), and PSG1^PE^ (**f**), or recombinant PSG1^293^ (**g**).

**Supplementary Figure 5**

**Fig. S5: Affinity chromatography single injections and controls. a** and **b** Mannose receptor (ManR) affinity chromatography elution profiles of PSG1^CHO^ and PSG1^293^ as single injections of duplicate measurements. **c** and **d** Asialoglycoprotein receptor (ASGR) affinity chromatography elution profiles of PSG1^CHO^ and PSG1^293^ as single injections. The ASGR gradient was optimized after the first run; therefore, the chromatogram (**c**) ends at 35 min. Both gradients show the same pattern: stronger interaction with PSG1^293^ than with PSG1^CHO^. **e** ManR affinity chromatography elution profiles of controls consisting of a monoclonal antibody (mAb) in duplicates and a blank (buffer) injection. **f** ASGR affinity chromatography elution profiles of Etanercept and neuraminidase-digested Etanercept (Etanercept-Neuraminidase) as controls and a blank (buffer) injection.

**Supplementary Figure 6**

**Fig. S6: Western blot analysis of recombinant PSG1 fractions after mannose receptor affinity chromatography. a** Western blot analysis of collected fractions using the BAP3 antibody to detect eluted recombinant PSG1. **b** and **c** Collection of the annotated peaks (A–D) from the mannose receptor affinity chromatography column, injecting PSG1^CHO^ (**b**) or PSG1^293^ (**c**).

**Supplementary Figure 7**

**Fig. S7: Specificity controls for labelled protein uptake in cells co-incubated with 200 µg/mL asialofetuin.** Quantitative analysis of Alexa Fluor 488 intensity in the cytoplasm, averaged per cell and shown per condition (mean ± SD). Welch's t-test showed no significant difference between the two groups.
